# Type II Renal Tubular Acidosis Secondary to Topiramate: A Review

**DOI:** 10.7759/cureus.3635

**Published:** 2018-11-26

**Authors:** Ankur Sinha, Phone Oo, Muhammad U Asghar, Hira A Cheema, Sanwal S Mehta, Joshua C Leinwand, Kalyana Janga

**Affiliations:** 1 Pulmonary and Critical Care, Maimonides Medical Center, Brooklyn, USA; 2 Department of Nephrology, Vassar Brothers Medical Center, poughkeepsie, USA; 3 Surgery, New York University Langone Medical Center, New York, USA; 4 Internal Medicine, Markham Stouffville Hospital, Markham, CAN; 5 Internal Medicine, Maimonides Medical Center, Brooklyn, USA; 6 Department of Nephrology, Maimonides Medical Center, New York, USA

**Keywords:** renal tubular acidosis, topiramate

## Abstract

Topiramate (TMP) is a broad-spectrum anticonvulsant drug used to treat a wide variety of seizure disorders, for migraine prophylaxis, and for many other indications. An important side effect of TMP is metabolic acidosis, which is mediated by renal tubular defects. TMP inhibits carbonic anhydrase, an enzyme that is necessary for acid handling in the proximal renal tubule. Patients can present with asymptomatic serum electrolyte derangements, acute change in mental status, hyperventilation, cardiac arrhythmias, or other sequelae of metabolic acidosis and associated respiratory compensation. If taken chronically, TMP can cause renal stone formation, bone mineralization defects, and several other effects secondary to changes in serum and urine pH and electrolytes. There is no well-studied way to prevent metabolic acidosis in patients taking TMP, but physicians should be vigilant when prescribing this drug to patients with the history of renal diseases and other comorbidities, and aware of this potential etiology of metabolic acidosis. We present a literature review of the underlying mechanisms involved in the development of renal tubular acidosis secondary to TMP and its clinical consequences.

## Introduction and background

Topiramate (TMP) is an anticonvulsant drug used to treat epilepsy in adults and children. According to American Academy of Neurology (AAN) guidelines, TMP can be used as initial therapy for newly diagnosed focal and mixed seizure disorders [1]; it is also used as monotherapy for refractory generalized tonic-clonic seizures and focal seizures in adults and children [2]. TMP has also been used for migraine prophylaxis, weight reduction, among other indications [3-5]. TMP is primarily excreted through the kidney and can cause renal tubular acid-base disturbances. With the wide and increasing use of this drug and its potential for renal side effects, it is imperative to consider a patient’s comorbidities before prescribing TMP and to be aware of possible drug toxicities when evaluating electrolyte derangements in patients taking this drug.

**Figure 1:**
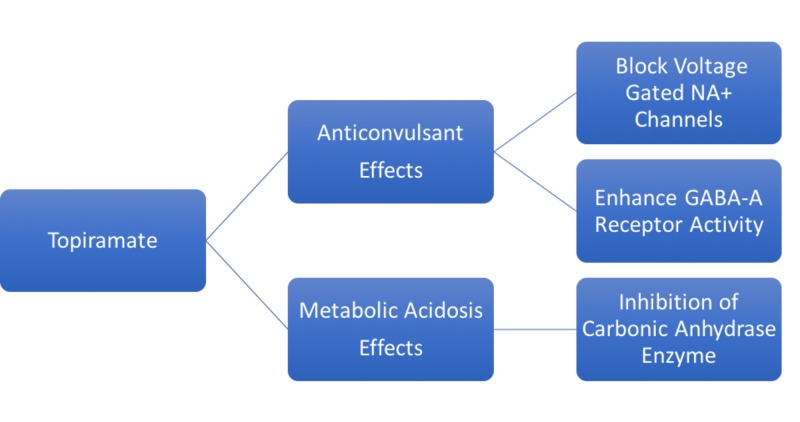
Mechanisms of Action of Topiramate.

TMP is approved by the US Food and Drug Administration (FDA) for use in adults and children two years of age or older for epilepsy monotherapy or adjunctive therapy [3]. It is also approved for migraine prophylaxis [4], and for weight reduction in combination with phentermine [5]. Most patients achieve 90% of the maximum plasma concentration within two hours of oral administration [6]. TMP is unlikely to displace highly protein-bound drugs in the plasma, hence limiting its drug interactions. The predominant route of drug elimination is renal excretion, accounting for about 51% of TMP elimination [6]. The therapeutic activities and side effects of TMP are mediated by several different mechanisms of action (Figure 1).

## Review

Metabolic acidosis occurs when there is an imbalance between the body’s production of H+ ions, and the kidneys’ ability to excrete H+ ions and resorb HCO_3^-^_. Metabolic acidosis is classified as either high anion gap (caused by increased production or ingestion of acid and impaired renal acid excretion) or normal anion gap (caused by the loss of bicarbonate from the kidney or gastrointestinal tract).

**Figure 2:**
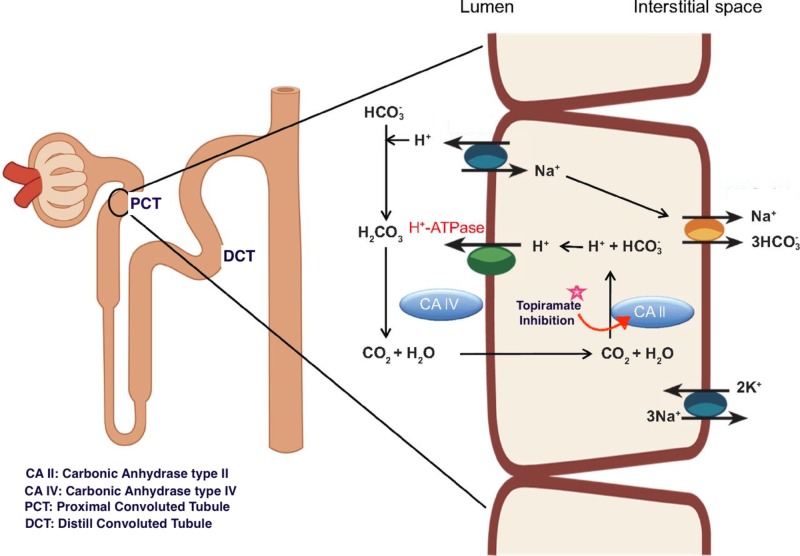
Development of Proximal Renal Tubular Acidosis.

Renal tubular defects can result in metabolic acidosis due to impaired renal H+ excretion or HCO_3^-^_ resorption. Carbonic anhydrase (CA) is an enzyme that catalyzes the formation of H+ and HCO_3^-^_ from CO_2_ and H_2_O, which is necessary for renal excretion of H+ and resorption of HCO_3^-^_ in the proximal convoluted tubule (PCT). Of the various types of CA, CA type II (CA-II) predominates in the human kidney, comprising about 95% of the total CA, while the remaining 5% consists of CA-IV and CA-XIII [7]. Supuran et al. reported that TMP is a potent inhibitor of CA-II and CA-XIII, and a medium potency inhibitor of CA-IV [8]. Because of its CA-inhibitory activity, especially against CA-II, TMP can impair H+ excretion and HCO_3^-^_ absorption in the PCT, leading to increased delivery of HCO_3^-^_ in the distal portion of the nephron and induce a normal anion gap metabolic acidosis (Figure 2).

Distal (Type 1) renal tubular acidosis (RTA) is characterized by impaired acid secretion from the collecting tubules, leading to an inability to secrete acid, and resulting in progressive H+ ion accumulation which manifests clinically as decreased serum HCO_3^-^_ [9]. Proximal (Type 2) RTA is caused by an inability to reabsorb HCO_3^-^_ from the PCT. Since the PCT reabsorbs 85 to 90% of the filtered load of HCO_3^-^_, inhibition of CA-II by TMP can lead to Type 2 RTA. Type 2 RTA can also result from Fanconi syndrome, a generalized resorption defect affecting HCO_3^-^_, glucose, phosphate, amino acids, and tubular proteins. The differences between proximal and distal RTA are shown in Table 1.

**Table 1:** Type 1 and Type 2 Renal Tubular Acidosis (RTA).

	Distal (Type 1) RTA	Proximal (Type 2) RTA	Study Ref.
Pathophysiology	Failure to secrete H^+ ^and reabsorb K^+ ^Ions	Failure to reabsorb HCO_3_	
Location	Collecting & distal tubules	Proximal Tubules	
Urine pH	> 6.0	<5.5	13
Urine HCO_3_^- ^	Normal	Increased	13
Fractional Excretion of HCO_3_^-^	<5%	>15%	13
Serum Potassium	Hypokalemia	Hypokalemia	

There are several reports in the literature of an association between TMP and hyperchloremic normal anion gap metabolic acidosis with an alkaline urine and positive urine anion gap (due to increased urine HCO_3^-^_) [10, 11]. In many cases, patients may be asymptomatic. Burmeister et al. [12] reported a case of severe metabolic acidosis in a 46-year-old woman who was prescribed TMP, 100 mg per day for three months; Ozer and Altunkaya described a similar presentation in a 58-year-old man [11]. Other studies have demonstrated the role of TMP in lowering plasma HCO_3^-^_; in a cross-sectional study, Welch et al. compared 32 TMP-treated subjects and 50 healthy volunteers and concluded that TMP-treated subjects had significantly lower serum total carbon dioxide content [13]. In another study, there was a 2.0 kg mean decline in weight after three months’ use of TMP [14].

Metabolic acidosis can present with complications due to respiratory compensation, notably hyperventilation, fatigue, altered mental status; more severe cases can lead to cardiac arrhythmia and coma. Stowe et al. [15] described a case of TMP-induced metabolic acidosis in a 20-year-old man presenting with disorientation, somnolence, agitation, and headache for two weeks.

Renal tubular acidosis caused by TMP can lead to decreased urine citrate concentration; hypocitraturia combined with decreased urine acidification in the distal convoluted tubules can contribute to the formation of calcium phosphate stones [16, 17]. The risk of the renal stone formation increases to 10 folds with the use of TMP. Warner et al. [18] in a study involving four subjects, reported that urinary citrate levels decreased significantly and rapidly after the start of TMP therapy and continued to decrease with escalating doses. Other common sides in adults are related to central nervous system (CNS) including paresthesia, fatigue, dizziness, somnolence, and mood symptoms [19]. TMP is also associated with oligohydrosis due to its inhibition of carbonic anhydrase within the sweat glands leading to impaired seat rate which is associated with heat intolerance and hyperthermia particularly in children [20].

There are no evidence-based or universally accepted management strategies to treat RTA secondary to TMP. TMP discontinuation should be considered in patients with persistent severe RTA. In some reports, normalization of mental status in patients with TMP-induced metabolic acidosis took 48 hours after the discontinuation of TMP [17]. Treatment with an alkali such as sodium bicarbonate or potassium citrate and citric acid can be used to restore normal serum HCO_3^-^_ in patients with RTA. This can also decrease urinary calcium excretion and increase urinary citrate excretion which can prevent renal stone formation and improve bone disease in adults [21]. Adequate hydration whilst using TMP should be encouraged as it can reduce the risk of developing renal stones.

## Conclusions

Topiramate has been demonstrated to be a potent inhibitor of some carbonic anhydrase isoenzymes, which can result in the development of type 2 renal tubular acidosis, normal anion gap metabolic acidosis, and nephrolithiasis as side effects from its use. Clinicians must remain vigilant to the clinical manifestations of metabolic acidosis, and aware of this possible etiology.
