# Correction: Type II Renal Tubular Acidosis Secondary to Topiramate: A Review

**DOI:** 10.7759/cureus.c17

**Published:** 2019-02-04

**Authors:** Ankur Sinha, Phone Oo, Muhammad U Asghar, Hira A Cheema, Sanwal S Mehta, Joshua C Leinwand, Kalyana Janga

**Affiliations:** 1 Pulmonary and Critical Care, Maimonides Medical Center, Brooklyn, USA; 2 Department of Nephrology, Vassar Brothers Medical Center, poughkeepsie, USA; 3 Surgery, New York University Langone Medical Center, New York, USA; 4 Internal Medicine, Markham Stouffville Hospital, Markham, CAN; 5 Internal Medicine, Maimonides Medical Center, Brooklyn, USA; 6 Department of Nephrology, Maimonides Medical Center, New York, USA

Ankur Sinha, Phone Oo and Kalyana Janga were added to the article as authors as they were erroneously excluded from the original submission. It has been confirmed that all three participated in the case and all authors are in agreement regarding their inclusion.

 

